# Impacts of visitors on female pheasants in pheasantry, Haripur, Pakistan

**DOI:** 10.7717/peerj.18031

**Published:** 2024-09-18

**Authors:** Nehafta Bibi, Binqiang Li, Habiba Zaffar, Muqaddas Salahuddin, Romana Gul, Zafeer Saqib, Rehana Khan, Fiza Mazhar, Aymen Shehzadi, Laraib Fiaz, Muneeba Naseer, Xu Luo

**Affiliations:** 1 Key Laboratory for Conserving Wildlife with Small Populations in Universities of Yunnan Province/College of Forestry, Southwest Forestry University, Kunming, China; 2 Department of Zoology, Government Girls Degree College #1 Mansehra, Mansehra, Pakistan; 3 GIS and Eco-Informatics Laboratory, Department of Environmental Science (DES), International Islamic University, Islamabad, Asia, Pakistan; 4 Department of Physics, Higher Education Colleges, Government of Khyber Pakhtunkhwa Pakistan, Nowshera, Asia, Pakistan; 5 College of Biological Sciences and Food Engineering, Southwest Forestry University, Kunming, China

**Keywords:** Human-wildlife interactions, Pheasantry, Visitors impact, Conservation management, Avian behavior, Behavioral adaptation, Captive bird welfare

## Abstract

**Background:**

The interaction between visitors and captive birds is complex, with a potential impact on bird’s behavior and welfare. Understanding this interaction is essential for effective conservation and management.

**Methods:**

We conducted a study at the University of Haripur’s pheasantry in Khyber Pakhtunkhwa, Pakistan to investigate the effects of visitor numbers, duration of visitor presence, and climatic factors on the behavior of female pheasants. We observed the state and events of feeding, hiding, and moving behaviors of 16 randomly selected individuals from five species.

**Results:**

The mixed-effects modeling results show that visitors (VT), visitors’ presence duration (VPD), and temperature (TP), significantly influence feeding events (*p* < 0.001), feeding duration (*p* < 0.001), hiding events (*p* < 0.001) and hiding duration of female pheasants (*p* < 0.001). The moving events of pheasants were also significantly affected by both VT and VPD (VT: *p* = 0.002, VPD: *p* < 0.001). Moreover, under high visitor conditions, the impact of VPD on the behavior of female pheasants was more pronounced (*p* < 0.001). Additionally, our result reveals that different species of pheasants exhibit varying sensitivities to human factors and climatic factors. For instance, the two species of female pheasants with the highest feeding and hiding events were the Green pheasant (*Phasianus versicolor*) and the Ring-necked pheasant (*Phasianus colchicus*). While hiding duration of female Green pheasants, female Golden pheasants (*Chrysolophus pictus*), and female Silver pheasants (*Lophura nycthemera*) was longer than those of others. The mean number of moving events was highest in females of Ring-necked, followed by Golden pheasants. The female Indian peafowl (*Pavo cristatus*) and female Silver pheasants were the birds with the longest moving duration.

**Conclusion:**

Our findings highlight the necessity for customized management strategies, to lessen the effects of human disturbances in pheasantries. For a thorough understanding of these interactions, more studies involving larger sample sizes and a wider variety of species are advised.

## Introduction

Bird species are especially vulnerable to human disturbance due to close relationships between their habitats, populations, behaviors, and the environment (Kerr & Currie, 1995; Jetz, Wilcove & Dobson, 2007; Tuomainen & Candolin, 2011). Human disturbances, including habitat loss, noise pollution, recreational activities, *etc*., can drastically impact the behavior, fitness, and reproductive success of birds (Martínez-Abraín et al., 2010; Warrington et al., 2022). These disturbances can disrupt breeding habits, resulting in lower reproductive yield and population declines (French et al., 2011). For instance, disturbances caused by tourists or other visitors in protected areas can induce stress responses in birds affecting their energy expenditure, territorial behavior, and foraging habits (Kangas et al., 2010). Understanding these complex interactions between humans and wildlife is crucial in mitigating the impacts of human disturbances on avian populations.

Within pheasantries, enclosures of captive pheasants can be particularly eye-catching due to the distinctive and beautiful look of many species (Fuller & Garson, 2000). Pheasantries serve as vital resources for research, education, recreation, and the preservation of genetic diversity with the use of *ex-situ* conservation, reintroductions, and restocking initiatives (World Pheasant Association, 2009). Pheasantries also offer a unique chance to study how different pheasant species react behaviorally to human disturbances as they provide a semi-captive environment where interactions between humans and captive pheasants are inevitable (Hauptmanova, Maly & Literak, 2006; Price, 2008; World Pheasant Association, 2009). However, the relationship between the caged animals and the visitors is complex, and both parties can have a significant impact on the other (Sherwen & Hemsworth, 2019; Collins, McKeown & O’Riordan, 2023). Visitors may experience feelings of happiness, relaxation, excitement, interest, and empathy for the animals during their visits while some captive animals may experience higher levels of stress compared to their wild counterparts (Alatossava, 2022; Woods, Eyer & Miller, 2022).

Zoo animals have been shown to have either negative, neutral, or positive effects on visitors. For instance, visitors have been observed to provoke fear in little penguins (*Eudyptula minor*) (Chiew et al., 2019), while no direct visitor effect has been observed in the case of a pair of hornbills (Rose, Scales & Brereton, 2020). Several species, including the African spoonbill (*Platalea alba*), Red-legged seriema (*Cariama cristata*), Inca tern (*Larosterna inca*), Boat-billed heron (*Cochlearius cochlearius*), Black-bellied whistling duck (*Dendrocygna autumnalis*), and Buff-banded rail (*Hypotaenidia philippensis*), were found to adapt to human presence and exhibited no discernible changes in their observed behaviors (Blanchett, Finegan & Atkinson, 2020). Certain captive bird species including the Demoiselle crane (*Grus virgo*), Helmeted guineafowl (*Numida meleagris*), and Hottentot teal (*Spatula hottentota*) showed avoidance behavior by moving away from the habitat zone in the presence of visitors. However, Sunbittern (*Eurypyga helias*) employed vegetation cover more frequently when visitors number was high (Blanchett, Finegan & Atkinson, 2020). Bird’s preference for sheltered areas could be an effort to hide from visitors, which could interfere with their usual behavioral patterns (Morgan & Tromborg, 2007). The probable explanation of these behavioral changes is the theory of trade-off, which states that when individuals spend more time engaging in one behavioral activity, this must be counterbalanced by a comparable drop in at least one other behavioral activity (Favreau et al., 2014).

Animal behavior is widely used to evaluate the welfare of zoo animals and how well captive animals are perceived to be functioning in their current circumstances (Binding et al., 2020). Usually, the questions concerning animal behavior determine whether or not we should record it as events or as states. Events happen immediately and then normally estimated by the frequency, whereas states last for a sizable portion of time and are estimated by the duration of time spent on a given activity (Rose et al., 2022; Altmann, 1974). We chose to document both estimates of behavior to fully grasp the impact of visitors (Steinbrecher et al., 2023). In the present study, we tested whether the number of visitors, visitor’s presence duration, and climatic factors influence the behavior of these caged female pheasants. For this question, we predicted that when the number of visitors and visitor’s presence time increase, 1) feeding events or feeding duration will no matter decrease, 2) hiding events or hiding duration will increase, and 3) moving events or moving duration will decrease.

By answering this research question, we pave the way for the development of evidence-based management strategies for pheasantries and other semi-captive environments, thus aiding in the conservation of bird species and their habitats.

## Materials and Methods

### Study site and design

The current study was conducted at the pheasantry of the University of Haripur, Khyber Pakhtunkhwa, Pakistan (approved by the Research Ethics/Bioethics committee of the University of Haripur under approval number UOH/DASR/2024/2005). The Department of Forestry at the University of Haripur created a pheasantry on the campus and it covers an area of 8,500 m^2^. It contains eight species of pheasants *i.e*., the Indian peafowl (*Pavo cristatus*), Golden pheasant (*Chrysolophus pictus*), Ring-necked pheasant (*Phasianus colchicus*), Silver pheasant *(Lophura nycthemera*), Reeves’s pheasant (*Syrmaticus reevesii*), Green pheasant (*Phasianus versicolor*), Lady Amherst’s pheasant (*Chrysolophus amherstiae*) and Kalij Pheasant (*Lophura leucomelanos*). Every species of pheasant was kept in separate enclosures, each measuring 3.04 × 6.09 × 3.04 m (length × width × height).

The interior layout of the enclosures makes the most use of the available space, with areas designated for perching, nesting, feeding, and hiding. The arrangement of the food bowls minimized spillage while facilitating easy access to the food. The pheasant’s feeding habits were taken into consideration when selecting the bowl’s height and size to ensure that they could easily get their food. The top of every enclosure in the pheasantry and also those we used were covered with a strong steel sheet that protects the bird’s food and water from the rain and heat. In addition, the enclosure’s fence was painted green, which enhances its appearance and serves several practical purposes. For instance, it provides a vital line of defense for the birds by acting as a deterrent to predators. Moreover, the green color provides camouflage and lessens visibility to possible threats because it blends in with the surrounding vegetation.

This vegetation inside the cages includes neem (*Azadirachta indica*), bottlebrush (*Melaleuca viminalis*), eucalyptus (*Eucalyptus camaldulensis*), and chir pine (*Pinus roxburghii*). Both the Australian native eucalyptus and the Himalayan native chir pine offer shade and help to control the enclosure’s temperature. Naturally occurring in Australia, bottlebrush serves as a component of the natural habitat and adds visual interest. India’s native neem adds more foliage and enhances the overall diversity of the habitat. In addition to improving the pheasants’ living conditions, these plants provide them with natural aesthetics and hiding places. In addition to being a research facility, the pheasantry is a popular tourist destination that draws staff, students, and their friends from nearby areas (Fig. S1).

### Subject

The study was conducted from 22 March to 8 April 2022 (15 days, except weekends). By laying eggs and raising chicks, female pheasants make a significant contribution to population growth and reproduction. Therefore, only female individuals were chosen for observation. A random selection of five species (two individuals of Indian peafowl, four Ring-necked pheasants, two Silver pheasants, two Golden pheasants, and six green pheasants) was made from a total pool of eight pheasant species following random sampling methodology of Crockett & Ha (2010). Individuals of each species were housed in separate enclosures, with females and males kept together. The composition of each enclosure was as follows: one male and two Indian peafowl females, one male and four Ring-necked female pheasants, one male and two Silver female pheasants, one male and two Golden female pheasants, and one male and six Green female pheasants. Sex identification was performed using plumage characteristics (Kayvanfar, Aliabadian & Ghasempouri, 2015). Continuous focal sampling was used from 10 am–5 pm to measure both the state and events of feeding, hiding, and moving behavior of a focal individual. A single focal individual was selected for recording both state and events of feeding, hiding, and moving behavior (Cooper & Jordan, 2013; Bosholn & Anciães, 2022). This time was decided to align with the institution’s operating hours.

Data were collected using the camera trap (PC 900 HyperFire Professional IR) as it may be the most appropriate technique for monitoring the behavior of large, ground-dwelling pheasants (Fischer et al., 2017). We placed one camera in the upper corner of each cage to provide maximum coverage of the ground and to avoid obstructing the bird. Consequently, during the course of our research, the data represent observations of five birds in total. From the continuous recordings, we extracted six behavior estimates: feeding events, feeding duration, hiding events, moving events, and moving duration with 35 h per day and a total of 525 h (Steinbrecher et al., 2023). Our cameras record videos that last for 1 min.

In addition, five observers were placed on the lawn to manually record the state and events of feeding, hiding, and moving behaviors of the focal individual. They also recorded the number of visitors and the duration of their presence near each of the five cages. To lessen the possibility of any observer effects, the observers’ positions were hidden from the birds inside the cages.

An ethogram was developed and adopted from the previous studies (Blanchett, Finegan & Atkinson, 2020; Sherwen et al., 2015; Zapletal et al., 2011; Table 1). We counted the events of each behavior per hour before statistical analyses. We also used the sum of the total time duration of each behavior per hour for analysis (Steinbrecher et al., 2023). Environmental variables including hourly temperature and relative humidity were collected from the weather station of the city.

**Table 1:** Ethogram adopted from previous studies (Zapletal et al., 2011; Sherwen et al., 2015; Blanchett, Finegan & Atkinson, 2020).

Behavior	Description	State/Event behavior
Feeding	Food intake behavior, picking food from the food bowl with head lowered, and consumption of food.	Feeding event Feeding duration
Hiding	Being stationary in the bushes or moving towards bushes when perceiving or alarmed by any danger.	Hiding event Hiding duration
Moving	Walking back and forth in a set route with no apparent goal (neither towards the feeding bowl nor towards bushes), including walking with both heads upright and lowered.	Moving event Moving duration

### Statistical analysis

First, we divided the number of visitors into low, medium, and high quartiles to determine the dividing point. We calculated the first quartile (Q1, the value at 25% percentile), the second quartile (Q2, the median, the value at the 50th percentile), and the third quartile (Q3, the value at the 75th percentile). Specifically, we categorized visitor numbers as follows: low (<Q1, <3), middle (between Q1 and Q3, 3 to 9, inclusive), and high (greater than Q3, >9). Due to the existence of multilinearity between variables, we used Spearman correlation analysis to obtain the correlation coefficient between variables. There was a significant negative correlation between temperature and relative humidity, so we removed the relative humidity. This allowed us to obtain the individual effects and percentages of each variable’s contribution to the model.

In all data analysis, linear mixed-effects models were utilized to examine the effects of human and climatic factors on the behavior of female pheasants. The variables ‘visitors number,’ ‘visitor presence duration,’ and ‘temperature’ were log-transformed and treated as fixed factors, while the date of observation and the species were considered random factors. The Akaike Information Criterion (AIC) was applied to select the most optimal model, with a lower AIC indicating a better fit. Models with a ΔAICc of less than two were deemed the best. To determine the individual effects and the percentage contributions of human factors and climatic variables to the model, a hierarchical partitioning method was employed.

Furthermore, to investigate the impact of visitor categories (high, medium, low), linear mixed-effects models were also fitted, following the same analytical steps as the overall data analysis (which did not categorize visitor numbers). To discern the differential responses of five species of pheasants to human and climatic factors, multiple comparisons were conducted using linear mixed-effects models. The fixed factors included ‘species,’ ‘visitors,’ ‘visitor presence duration,’ and ‘temperature,’ with the date of observation as a random factor. The Tukey’s Honestly Significant Difference (Tukey’s HSD) test was applied for inter-species multiple comparisons.

All analyses were conducted in R 4.2.2 (R Core Team, 2022). We utilized the ‘lme4’ package for mixed-effects modeling (Bates et al., 2015), the ‘MuMIn’ package for model selection (Barton, 2017), the ‘glmm.hp’ package for hierarchical partitioning (Lai & Tang, 2024), and the ‘emmeans’ and ‘multcomp’ packages for inter-species multiple comparisons (Lenth, 2021; Hothorn et al., 2022).

## Results

Results from the mixed-effects modeling indicate that visitors (VT), visitor’s presence duration (VPD), and temperature (TP) significantly influence the feeding event (*p* < 0.001), feeding duration (*p* < 0.001), hiding events (*p* < 0.001), and hiding duration of female pheasants (*p* < 0.001) (Table 2). The visitor’s presence duration exerts the most substantial impact on pheasant behavior. Both VT and VPD also significantly affect the moving events of pheasants, with VPD having the greatest influence on these activities (VT: *p* = 0.002, VPD: *p* < 0.001). When visitors number was low to medium, TP had the most significant impact on pheasant behavior. However, in high visitor conditions, the influence of VPD on pheasant behavior becomes more pronounced (*p* < 0.001; Table 2). Additionally, our result reveals that different species of pheasants exhibit varying sensitivities to human factors and climatic factors (Table 3).

**Table 2:** Linear mixed-effects analysis of human factors and climatic factors on pheasant behavior.

	Predictor	Estimate	SE	df	t value	*p* value	I. perc (%)
**All data**							
Feeding events	VT	−0.261	0.029	514.89	−8.969	*p* < 0.001	30.08
	VPD	−0.442	0.032	505.55	−13.610	*p* < 0.001	44.86
	TP	−1.184	0.163	496.95	−7.252	*p* < 0.001	25.06
Feeding duration	VT	−0.185	0.027	516.29	−6.818	*p* < 0.001	29.07
	VPD	−0.369	0.030	506.24	−12.145	*p* < 0.001	52.52
	TP	−0.608	0.152	477.01	−4.003	*p* < 0.001	18.41
Hiding events	VT	0.290	0.032	498.65	9.069	*p* < 0.001	33.84
	VPD	0.304	0.036	504.88	8.427	*p* < 0.001	26.84
	TP	1.951	0.183	514.48	10.672	*p* < 0.001	39.32
Hiding duration	VT	0.127	0.021	502.88	5.962	*p* < 0.001	25.86
	VPD	0.216	0.024	504.85	9.051	*p* < 0.001	36.1
	TP	1.045	0.121	514.61	8.624	*p* < 0.001	38.04
Moving events	VT	−0.132	0.043	510.89	−3.054	0.002	40.57
	VPD	−0.225	0.049	510.93	−4.584	*p* < 0.001	59.43
**Low (number of visitors)**							
Feeding events	VPD	−0.378	0.130	70.63	−2.900	0.0049	40.7
	TP	−0.444	0.096	68.54	−4.614	*p* < 0.001	45.83
	VT	−0.809	0.424	11.80	−1.908	0.081	13.47
Feeding duration	VPD	−0.246	0.096	71.67	−2.561	0.013	41.35
	TP	−0.786	0.413	26.32	−1.904	0.068	58.65
Hiding events	VPD	0.342	0.110	79.23	3.114	0.003	
Hiding duration	VPD	0.257	0.086	75.39	2.982	0.003	35.06
	TP	0.785	0.381	29.36	2.060	0.048	64.94
**Medium (number of visitors)**							
Feeding events	VPD	−0.292	0.042	240.78	−6.973	*p* < 0.001	44.01
	TP	−1.262	0.159	246.50	−7.917	*p* < 0.001	55.99
Feeding duration	VT	−0.215	0.056	242.02	−3.807	*p* < 0.001	18.67
	VPD	−0.227	0.035	244.03	−6.459	*p* < 0.001	58.07
	TP	−0.417	0.135	241.89	−3.086	0.002	23.26
Hiding events	VT	0.582	0.090	242.04	6.440	*p* < 0.001	33.15
	VPD	0.192	0.057	243.34	3.400	*p* < 0.001	13.22
	TP	1.607	0.219	246.57	7.334	*p* < 0.001	53.63
Hiding duration	VPD	0.133	0.036	241.94	3.693	*p* < 0.001	28.51
	TP	0.922	0.137	246.53	6.722	*p* < 0.001	71.49
Moving duration	VT	0.202	0.055	242.32	3.694	*p* < 0.001	
**High (number of visitors)**							
Feeding events	VT	−0.812	0.206	130.82	−3.946	*p* < 0.001	34.24
	VPD	−0.542	0.102	133.79	−5.297	*p* < 0.001	57.13
	TP	−1.655	0.773	65.78	−2.140	0.036	8.63
Feeding duration	VT	−0.413	0.200	137.11	−2.071	0.04	28.36
	VPD	−0.340	0.100	138.68	−3.405	*p* < 0.001	71.64
Hiding events	VPD	0.244	0.063	130.98	3.888	*p* < 0.001	68.7
	TP	1.198	0.463	36.09	2.587	0.014	31.3
Hiding duration	VPD	0.210	0.052	142.56	4.023	*p* < 0.001	
Moving events	VPD	−0.396	0.143	137.51	−2.766	0.006	85.43
	TP	−0.952	0.998	46.61	−0.954	0.345	14.57
Moving duration	VT	0.337	0.141	137.40	2.395	0.018	51.17
	TP	0.952	0.486	47.90	1.958	0.056	48.83

**Table 3:** Linear mixed-effects analysis of species differences towards the effects of human factors and climatic factors on pheasant behavior.

Species	Mean ± SD	emmean	SE	df	Lower. CL	Upper.CL	Significant group
**Feeding events**							
Silver pheasant	8.52 ± 5.45	1.78	0.0558	47.3	1.77	1.78	a
Red-golden	8.05 ± 5.93	1.87	0.0545	43.5	1.87	1.88	a
Indian Peafowl	7.61 ± 4.26	1.9	0.0545	43.5	1.9	1.91	a
Green pheasant	15.39 ± 3.87	2.59	0.0549	44.4	2.58	2.59	b
Ring-necked	14.92 ± 5.34	2.74	0.0549	44.5	2.74	2.75	b
**Feeding duration**							
Red-golden	8.08 ± 4.78	1.93	0.0481	53	1.92	1.93	a
Silver pheasant	8.65 ± 3.79	1.94	0.0494	58.2	1.94	1.95	a
Indian Peafowl	8.25 ± 3.53	2.05	0.0481	53	2.05	2.05	a
Green pheasant	16.70 ± 4.01	2.7	0.0485	54.3	2.7	2.7	b
Ring-necked	17.09 ± 5.59	2.85	0.0485	54.5	2.84	2.85	b
**Hiding events**							
Indian Peafowl	13.59 ± 6.78	2.33	0.0673	33.3	2.32	2.33	a
Ring-necked	15.98 ± 8.13	2.48	0.0677	34	2.48	2.48	ab
Red-golden	16.88 ± 9.53	2.54	0.0673	33.3	2.54	2.55	b
Silver pheasant	13.22 ± 6.28	2.56	0.0686	35.8	2.56	2.57	bc
Green pheasant	14.79 ± 5.89	2.74	0.0677	33.9	2.73	2.74	c
**Hiding duration**							
Indian Peafowl	17.15 ± 6.83	2.72	0.0448	33	2.72	2.73	a
Ring-necked	19.31 ± 4.80	2.88	0.045	33.7	2.87	2.88	b
Silver pheasant	19.23 ± 7.82	2.93	0.0456	35.4	2.92	2.93	b
Red-golden	22.43 ± 10.01	2.95	0.0448	33	2.95	2.95	b
Green pheasant	19.17 ± 4.12	2.98	0.045	33.6	2.98	2.99	b
**Moving events**							
Silver pheasant	10.90 ± 6.98	2.08	0.073	114	2.07	2.08	a
Indian Peafowl	11.31 ± 7.39	2.19	0.0706	103	2.18	2.19	ab
Green pheasant	12.52 ± 4.62	2.42	0.0712	105	2.41	2.42	bc
Red-golden	15.50 ± 9.08	2.52	0.0706	103	2.52	2.53	c
Ring-necked	14.21 ± 6.28	2.59	0.0713	106	2.59	2.6	c
**Moving duration**							
Ring-necked	20.50 ± 5.10	2.97	0.0472	33.7	2.97	2.98	a
Green pheasant	21.20 ± 4.46	3.05	0.0472	33.6	3.04	3.05	a
Red-golden	25.39 ± 11.47	3.07	0.047	33	3.06	3.07	a
Indian Peafowl	36.17 ± 4.83	3.23	0.047	33	3.23	3.24	b
Silver pheasant	32.10 ± 5.31	3.48	0.0478	35.4	3.48	3.48	c

Results of the species-wise analysis show that in terms of feeding events, the female Green pheasants had the highest mean followed by female Ring-necked pheasants, female Indian peafowl, female Golden pheasants, and female Silver pheasants. The longest feeding durations were observed in female Ring-necked pheasants and female Green pheasants, while female Silver pheasants, female Golden pheasants, and female Indian peafowl displayed marginally shorter durations. Female Green pheasants and female Silver pheasants showed the greatest number of hiding events, followed by female Ring-necked, female Golden, and female Indian peafowl. Furthermore, the hiding duration of female Green pheasants, female Golden pheasants, and female Silver pheasants was longer than those of female Ring-necked pheasants and female Indian peafowl. The mean number of moving events was the highest in female Ring-necked, followed by female Golden pheasants. The female Indian peafowl and female Silver pheasants were the birds with the longest moving duration (Table 3).

## Discussion

The results of our investigation demonstrate that visitor, visitor presence duration, and temperature have a major influence on the behavior of captive female pheasants. We found that feeding events, feeding duration, hiding events, and hiding duration were all impacted by the visitor’s presence duration; this effect was more noticeable under high visitor conditions. We may gain a deeper understanding of these dynamics by comparing our results with those of other studies and finding both consistencies and discrepancies.

Even though zoos are essential for scientific research, teaching, and conservation, Woods, Eyer & Miller (2022) found that visitors may be detrimental to the captive animals. Yet Blanchett, Finegan & Atkinson (2020) suggest that visitors may have neutral, negative, or positive effects on the behavior of captive birds. Our findings are consistent with earlier studies showing that visitors have a major influence on the behavior of captive animals (Sherwen & Hemsworth, 2019; Rose, Scales & Brereton, 2020). Our findings also align with Rose et al. (2022), who has shown that in conjunction with a change in sound environment, visitor presence can also change bird behavior. In contrast, some studies found no significant effects of visitors on certain bird species (Collins et al., 2016). Certain species might react to visitors by becoming more active and gregarious, while others might show signs of stress or avoidance (Price, 2008; Woods, Eyer & Miller, 2022). Alatossava (2022) has brought attention to the possible stress that visitors may cause captive birds, and this stress may have an impact on the birds’ behavior and general health. This demonstrates the difficulty in maintaining mixed-species exhibits and the necessity of close observation and modification of management strategies in response to the unique requirements of each species (Hauptmanova, Maly & Literak, 2006). For instance, interactions between visitors and captive animals can be intense and irregularly arranged, which may affect the animals’ behavior and general well-being (Sherwen & Hemsworth, 2019; Rose, Scales & Brereton, 2020). Our results also demonstrate that temperature has a major impact on bird behavior, which is consistent with earlier research (Rose, Scales & Brereton, 2020). This finding is also consistent with Goodenough et al. (2019) and Kidd, Rose & Ford (2022) who found that weather and time of day can have a bigger impact on zoo animal behavior. This highlights that to maintain the welfare of pheasants, management must take great care to manage human and climatic factors.

Likewise, we found variation in the response of different female pheasants towards human factors and climatic factors. For instance, female Green pheasants and female Ring-necked showed an increase in both events and state of feeding and hiding. These behavioral variations between female pheasant species highlight how crucial it is to take into account each species’ reaction to human factors and climatic factors (Crockett & Ha, 2010). These behavioral variations also demonstrate the importance of species-specific adaptations to human factors and climatic factors. For example, higher feeding and hiding states and events in female Green pheasants and female Ring-necked pheasants than other species may be an adaptation to visitors (Hauptmanova, Maly & Literak, 2006). Research conducted by Sherwen et al. (2015) has demonstrated that little penguins also display comparable increases in hiding behavior and distance from observer areas. Additionally, in the case of Humboldt penguins (*Spheniscus humboldti*), it was found that their ability for habituation became low, yet sensitivity to human activities increased (Mendes et al., 2020). This highlights the importance of acknowledging in management guidelines that even closely related species may respond differently to human presence.

Based on our research, two points should be considered in the future study. The enclosures available at the pheasantry in the University of Haripur, which have been built for decades, may not fully reflect the natural habitat in complexity and heterogeneity. The responding behavior of these pheasants may alter in their natural habitat or in larger enclosures that provide more space, as previous studies have shown (Rose, Brereton & Croft, 2018; de Azevedo et al., 2023). The second important point is the group composition in each enclosure, particularly the number of females. Females may experience less stress in larger groups than in smaller groups leading to varied behavioral responses towards visitors (Leone & Estevez, 2008; Hopper, 2021). This highlights the significance of the careful creation of enclosures that provide enough space and satisfy the social and behavioral demands of the species.

## Conclusion

In conclusion, our research shows that visitors, duration of visitor’s presence, and temperature, all have a substantial impact on the behavior of captive female pheasants. We found that the presence of visitors affected feeding events, feeding duration, hiding events, and hiding duration with the impacts being more noticeable during high visitor conditions. Our results are consistent with earlier studies showing that visitors can affect the behavior of caged animals, though species-specific differences exist in the responses. This highlights the significance of customized management approaches in zoos. To understand the variables causing behavioral changes in animals kept in captivity, more research is required.

## Supplemental Information

10.7717/peerj.18031/supp-1 Supplemental Information 1 Codes used for analysis.

10.7717/peerj.18031/supp-2 Supplemental Information 2 Supplementary material.

10.7717/peerj.18031/supp-3 Supplemental Information 3 Data used in analysis.

## Additional Information and Declarations

### Data Availability

The following information was supplied regarding data availability: The data and code used for analysis are available in the Supplemental Files.
