# Supplementary material for: Impacts of visitors on female pheasants in pheasantry, Haripur, Pakistan
Source: PeerJ. 2024 Sep 18;12:e18031. doi: 10.7717/peerj.18031 (PMC11416092; doi:10.7717/peerj.18031)
Supplement: Supplemental Information 2 [file peerj-12-18031-s002.doc]

**Impacts of Visitors on Female Pheasants in Pheasantry, Haripur, Pakistan**

Nehafta Bibi^1,2^, Binqiang Li^1^, Habiba Zaffar^2^, Muqaddas^2^, Romana Gul^2^, Zafeer Saqib^3^, Rehana Khan^4^, Fiza Mazhar^2^, Aymen Shehzadi^2^, Laraib Fiaz^2^, Muneeba Naseer^2^, Xu Luo^1,5*^

^1^ Key Laboratory for Conserving Wildlife with Small Populations in Universities of Yunnan Province / College of Forestry, Southwest Forestry University, Kunming 650224, China.

^2^ Department of Zoology, Government Girls Degree College #1 Mansehra 21300, Khyber Pakhtunkhwa Pakistan.

^3^ GIS and Eco-Informatics Laboratory (Room 131), Department of Environmental Science (DES) International Islamic University, Islamabad Pakistan.

^4^ Department of Physics, Higher education colleges, Govt. of Khyber Pakhtunkhwa Pakistan.

^5^ College of Biological Sciences and Food Engineering, Southwest Forestry University, Kunming 650224, China., Southwest Forestry University, Kunming 650224, China.

^*^Corresponding Author:

Xu Luo^1^

Email address: [luoxu@swfu.edu.cn](mailto:luoxu@swfu.edu.cn)


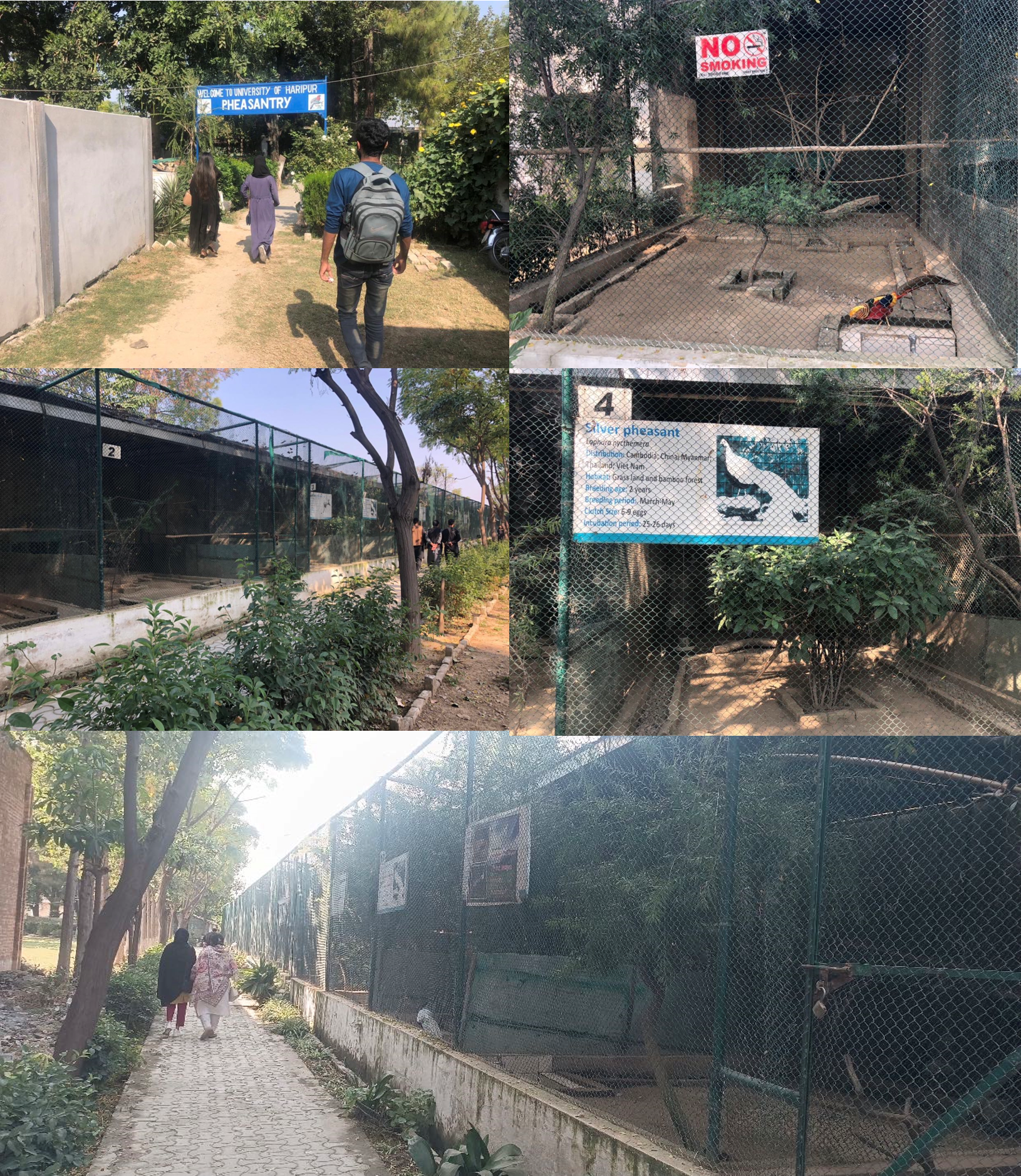


**Supplemental Figure 1**: Pheasantry at the University of Haripur, Haripur City Khyber Pakhtunkhwa Pakistan.

**Supplemental Table 1:** Spearman correlation analysis to obtain the correlation coefficient between human factors and climatic factors.

| **Variables** | **Visitors** | **Visitor’s presence duration** | **Temperature** | **Relative humidity** |
| --- | --- | --- | --- | --- |
| Visitors | 1 | 0.3670379 | 0.2439193 | -0.2866031 |
| Visitor’s presence duration | 0.3670379 | 1 | 0.2455348 | -0.3093949 |
| Temperature | 0.2439193 | 0.2455348 | 1 | -0.8404981 |
| Relative humidity | -0.2866031 | -0.3093949 | -0.8404981 | 1 |

**Supplemental Table 2:** Selection of the best model based on AIC.

|  | Predictor | df | AICc | delta | weight |
| --- | --- | --- | --- | --- | --- |
| **All data** |  |  |  |  |  |
| Feeding events | VPD+TP | 7 | 619.5 | 0 | 1 |
| Feeding duration | VPD+TP+VT | 7 | 549.2 | 0 | 0.996 |
| Hiding events | VPD+TP+VT | 7 | 724.8 | 0 | 1 |
| Hiding duration | VPD+TP+VT | 7 | 296.2 | 0 | 1 |
| Moving events | VPD+VT | 6 | 1069.3 | 0 | 0.555 |
| Moving duration | ~1 | 4 | 340.5 | 0 | 0.685 |
| **Low（number of visitors）** |  |  |  |  |  |
| Feeding events | VPD+TP+VT | 7 | 112.8 | 0 | 0.5 |
| Feeding duration | VPD+TP | 6 | 108.1 | 0 | 0.236 |
| Hiding events | VPD | 5 | 141.1 | 0 | 0.281 |
| Hiding duration | VPD+TP | 6 | 85.8 | 0 | 0.455 |
| Moving events | ~1 | 4 | 117.4 | 0 | 0.559 |
| Moving duration | ~1 | 4 | 94.2 | 0 | 0.692 |
| **Middle（number of visitors）** |  |  |  |  |  |
| Feeding events | VPD+TP | 6 | 214.4 | 0 | 0.611 |
| Feeding duration | VPD+TP+VT | 7 | 130.3 | 0 | 0.884 |
| Hiding events | VPD+TP+VT | 7 | 363.2 | 0 | 0.931 |
| Hiding duration | VPD+TP | 6 | 134.3 | 0 | 0.85 |
| Moving events | ~1 | 4 | 400.7 | 0 | 0.422 |
| Moving duration | VT | 5 | 113.7 | 0 | 0.669 |
| **High（number of visitors）** |  |  |  |  |  |
| Feeding events | VPD+TP+VT | 7 | 222.9 | 0 | 0.845 |
| Feeding duration | VPD+VT | 6 | 210.6 | 0 | 0.302 |
| Hiding events | VPD+TP | 6 | 85.9 | 0 | 0.449 |
| Hiding duration | VPD | 6 | 25.6 | 0 | 0.729 |
| Moving events | VPD+TP | 6 | 317.2 | 0 | 0.365 |
| Moving duration | TP+VT | 6 | 109.1 | 0 | 0.368 |
